# Clinical Follow-Up in Orofacial Clefts—Why Multidisciplinary Care Is the Key

**DOI:** 10.3390/jcm10040842

**Published:** 2021-02-18

**Authors:** Bernd Lethaus, Elisabeth Grau, Anita Kloss-Brandstätter, Luise Brauer, Rüdiger Zimmerer, Alexander K. Bartella, Sebastian Hahnel, Anna K. Sander

**Affiliations:** 1 Department of Oral and Maxillofacial Surgery, Leipzig University Medical Center, 04103 Leipzig, Germany; elisabeth.grau@gmx.de (E.G.); anita.kloss-brandstaettter@medizin.uni-leipzig.de (A.K.-B.); Ruediger.Zimmerer@medizin.uni-leipzig.de (R.Z.); alexander.bartella@medizin.uni-leipzig.de (A.K.B.); Anna.Sander@medizin.uni-leipzig.de (A.K.S.); 2 Department of Orthodontics, Leipzig University Medical Center, 04103 Leipzig, Germany; luise.brauer@medizin.uni-leipzig.de; 3 Department of Prosthetic Dentistry and Dental Materials Science, Leipzig University Medical Center, 04103 Leipzig, Germany; sebastian.hahnel@medzin.uni-leipzig.de

**Keywords:** cleft lip palate, multidisciplinary, team-oriented care, orofacial, syndrome, follow-up

## Abstract

(1) Background: Although most clinicians involved in the treatment of cleft patients agree upon the major importance of interdisciplinary cooperation and many protocols and concepts have been discussed in the literature, there is little evidence of the relevance of continuous interdisciplinary care. We aimed to objectify the type and number of therapeutic decisions resulting from an annual multidisciplinary follow-up. (2) Methods: We retrospectively analyzed the data of all 1126 patients followed up in the weekly consultation hours for cleft patients at university clinics in Leipzig for the years 2005–2020. We assessed the clinical data of every patient and specifically evaluated the treatment decisions taken at different points in time by the participating experts of different specialties. (3) Results: In total, 3470 consultations were included in the evaluation, and in 70% of those, a therapeutic recommendation was given. Each specialty showed certain time frames with intense treatment demand, which partially overlapped. Nearly all therapy recommendations were statistically attached to a certain age (*p* < 0.001). (4) Conclusions: There is an exceptionally high need for the interdisciplinary assessment of patients with cleft formation. Some developmental phases are of particular importance with regard to regular follow-up and initiation of different treatment protocols. The therapy and checkup of cleft patients should be concentrated in specialized centers.

## 1. Introduction

Due to their complex effects, patients with orofacial clefts face different problems in various fields. These include aesthetic appearance, speech-forming, hearing abilities, dental health, proper food intake, and psychological well-being. These findings suggest two major options for appropriate care in order to achieve favorable long-term outcomes. First is a team-oriented approach, including different specialties with each responsible for a specific problem, and second is regular follow-ups to implement or adapt any possible therapy. The American Cleft Palate–Craniofacial Association (ACPA) [1] and the European Committee for Standardization (CEN) [2] both emphasize the need for a team-oriented approach involving healthcare professionals. This idea has been evaluated by different groups who confirm the rationale [3,4,5]. Nonetheless, there is a shortage of hard data regarding the impact on the therapeutic process and the resulting benefits of multidisciplinary care.

In patients with orofacial clefts, the first year of life is typically the most intense in terms of the complete treatment sequence. Most centers begin immediately with presurgical orthodontic treatment and surgically address the lip and palate within the first year. This period marks the first therapeutic step in an ongoing treatment plan. It is common for an intense follow-up to be performed during this period by at least one healthcare professional. However, regular follow-ups should follow after this first intense treatment period to address upcoming problems and to initiate necessary procedures. To improve the patients’ comfort, they should be placed at the center of every treatment step, with the different healthcare specialists gathering around them rather than several consultations being organized with each specialist. A multi-team approach demands certain quantities of personal resources and is therefore easier to organize in larger centers.

The high investment of financial and personal resources should be justified by reliable data concerning the impact on the patients’ therapy course.

The aims of this study were to evaluate our patients with orofacial clefts who attended follow-up consultations within the last 15 years and to ascertain the type and number of therapy recommendations given by the different specialists. We want to provide information on the relevance of continuous multidisciplinary care by showing the number of treatment recommendations given by the different specialists at different times during patient growth. In the context of our therapy concept, this data should help identify periods with intense treatment needs and thus periods in which a controlled follow-up might be even more sensible.

The timely identification of further treatment requirements and the best therapy onset should result in an improvement in the patients’ therapy outcome and ultimately reduce healthcare costs.

## 2. Patients and Methods

In our tertiary medical care center, follow-up consultations were performed by a professional healthcare team, which consisted of maxillofacial surgeons, orthodontists, speech therapists, and Ear-Nose and Throat (ENT) physicians. Upon each visit, a patient was seen by all four specialties at the same appointment if the patient agreed. Newborn patients entered these follow-up consultations after the first year, when the surgical treatment of cleft lip and palate was completed. Patients and their families usually returned annually in their birth month. This has proven successful in facilitating a more consequential follow-up because it is easy to remember for the families. In addition to the newborn patients, this consultation hour was also open for other, usually adult, patients with orofacial clefts who had already been treated. In this retrospective study, we reviewed all charts from patients who attended these consultation hours for orofacial clefts between June 2005 and August 2020. All patients with a diagnosis of treated or untreated orofacial cleft between the ages of 0 and 56 years were included. Age, gender, cleft form, known existing syndrome, and the according therapy recommendations were recorded. Statistical analyses were performed with IBM SPSS (version 27; International Business Machines Corp., Armonk, NY, USA) and the free software environment. Pearson’s chi-squared test was applied to sets of unpaired categorical data to evaluate the likelihood that any observed difference between the sets was due to chance. Fisher’s exact test was used where sample sizes were small. The results of the statistical hypothesis tests were deemed significant when *p* < 0.05. With a sample size of 1126 patients, we formulated 3470 observations and 17 age groups, within which frequencies were compared, and we achieved >90% effectivity in detecting weak (w = 0.1) to mild effects (w = 0.3). For a graphical representation of data, again, the software environment R was applied.

## 3. Results

A total number of 3470 consultations, conducted with the 1126 patients who showed up for a regular follow-up, was included in the statistical evaluation. In total, 57% (*n* = 643) of the patients were male and 43% (*n* = 483) were female. The overall average of age was 10.20 years. Unilateral cleft lip and palate (33%) was found most frequently in one third of the patients, followed by isolated clefts of the hard and soft palate (29%) and unilateral cleft lip (9%) alone. In 6% of cases, the cleft occurred in the context of a syndromic disease. Seventeen different syndromes were described, all of which showed a relatively low incidence. A Pierre Robin sequence was observed in 32 cases.

**Figure 1:**
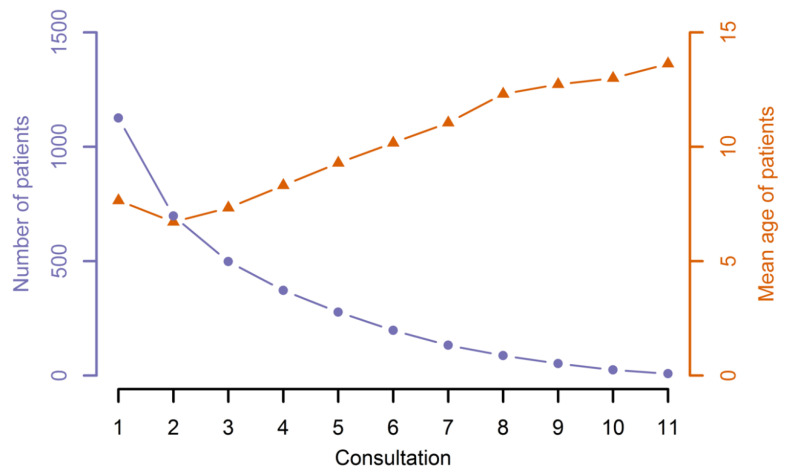
Follow-up visits of all patients within the observation period. The number of patients and their mean age according to the number of total consultations, which varied between 1 and 11, are shown.

**Figure 2:**
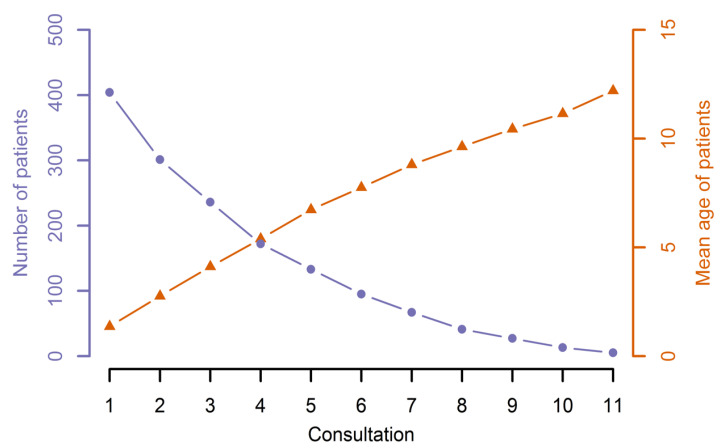
Follow-up visits and mean age of patients born during our investigation period.

The number of overall consultations varied between one and eleven. Figure 1 shows the number of patients attending the follow-up and the total number of attendances in the 15-year observation period. Although this period was set arbitrarily, the decreasing numbers of patients suggest irregular attendance. On average, every patient attended the follow-up three times during the studied period. This number could be skewed by the older patients who did not start their therapy in this 15-year interval or who showed up later with a specific concern. We therefore specifically considered patients born during the observation period and attending the follow-up for the first time (Figure 2). While we must again take into account the mere partial overlap in the investigation period, it becomes apparent that a substantial number of patients do not make use of the offer of a yearly follow-up.

Up until the children’s sixth birthday, there was a steady increase in the percentage of consultations in which treatment recommendations were given (Table 1). Up until the third birthday, recommendations were given in less than 50% of follow-up appointments, while for patients over four years old, a specific therapeutic intervention was recommended in 77% of cases. At the age of ten, some form of treatment was suggested in over 90% of the consultations.

**Figure 3:**
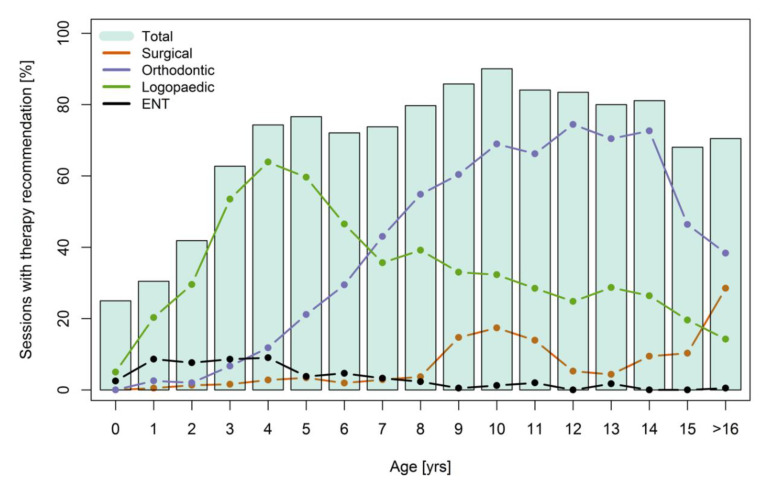
Proportion of consultations in which specific therapy recommendations were given by the participating specialists. ENT: Ear Nose Throat Physician.

Upon examination of the details of the recommendations given, a differing frequency distribution becomes apparent (Figure 3). The recommendation of speech therapy peaks at the age of three to six years, thus during the most relevant years for speech development and before school enrollment. At the age of four years, speech therapy was recommended to more than 60% of the patients. In 36% of all consultations, speech therapy was proposed.

The commencement or continuation of an orthodontic therapy was suggested slightly more often, in 37% of all consultations. The peak is reached notably later, as at twelve years of age, orthodontic therapy was suggested in 74% of examinations. Between the ages of eight and fourteen, orthodontic treatment was recommended in considerably more than 50% of all examinations.

The ENT treatment recommendation was observed mainly in the first four years, with a maximum of 9% at the age of four. The other treatment recommendations given were physiotherapy/occupational therapy (recommended in 4% of all cases, with a maximum of 9% of all patients seen at the age of five years) and dental treatment, recommended during 11% of all consultations.

The indication for surgical therapy was distributed more evenly, with peaks between 9 and 11 years of age and in patients 16 years and older. It is significant that primary surgical treatment (closure of cleft lip and palate) was not registered, as this was not considered in the follow-up consultation hours.

**Figure 4:**
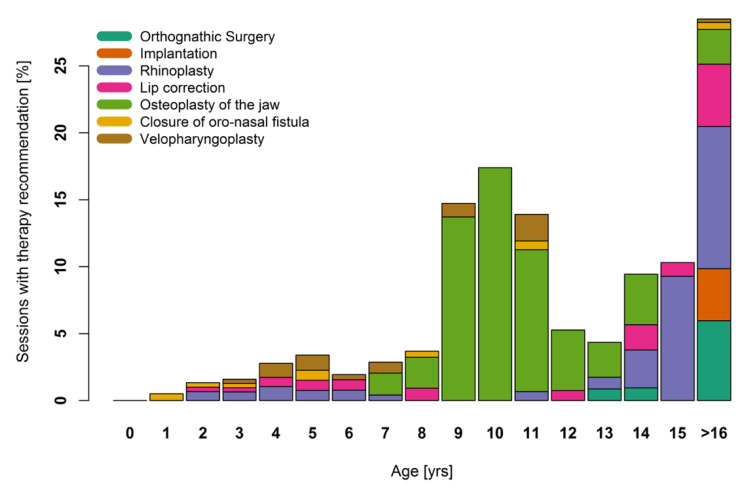
Proportion of different types of surgical therapeutic interventions per age.

The first peak reflects the recommendation of secondary osteoplasty of the jaw, while the increasing numbers in patients 16 years and older denote planned surgical interventions after the termination of growth (e.g., orthognathic surgery, and lip and nose correction). The other surgical therapies included are velopharyngoplasty (planned mainly between three and seven years of age), correction of the lip and tongue frenula, and closure of the oronasal fistula (featured in small numbers in all age groups) (Figure 4).

There was a significant association between age and most of the therapy recommendations, as can be seen in Table 2. In correlation analyses of age and velopharyngoplasty, correction of the frenula, and closure of the oronasal fistula, the numbers may have been too small to demonstrate statistical significance.

## 4. Discussion

A regular team approach to follow-up consultations with patients with orofacial clefts has been advocated by others. In this study, we were able to evaluate data concerning our own multidisciplinary consultation hour from the last 15 years in order to gain a broad view of its impact.

In the eyes of healthcare professionals, such a setting for annual follow-up seems favorable [6]. However, it does not automatically achieve acceptance by all patients and parents [7,8]. Interestingly, we found a rather low average number of visits per patient in contrast with other studies [9,10]. Canady and colleagues found that nearly half their patient group with orofacial clefts underwent more than ten consultation and that a third underwent between five and ten. This difference could be explained by the fact that this study concentrated on the follow-up consultations and left out the perioperative consultations, which are numerous in the first year yet are usually performed by the operating surgeon alone. Additionally, our data are skewed by the fact that all the patients in various stages of follow-up who were seen in the multidisciplinary consultation hour were included and that the observation period was chosen arbitrarily. A certain number of patients showed up only once with a specific concern, and this also explains the relatively high mean age of the patients. Still, focusing on patients born during the observation period results in a number of average visits per patient that is only slightly higher. These findings were unexpected and might be explained by a weariness after multiple contacts with the health care system that leads to patients omitting follow-up examinations when they themselves do not identify an acute problem. This could suggest that some patients would benefit from a recall program, calling them in on a yearly basis or at least during those stages of development with especially high treatment needs.

Three methods of follow-up are conceivable in the care of patients with orofacial clefts, as follows: I. regular follow-up by one healthcare professional, usually the surgeon, where the surgeon directs the patient to a distinct specialty if a problem is diagnosed or mentioned by the patient or their family; II. multiple follow-up consultations with every specialty independently from one another following a set scheme; and III. multidisciplinary follow-up, wherein the patient sees all the necessary specialties within one appointment [4,5,11,12]. The demand for personal and physical resources as well as the necessary degree of organization increases with each of these options. It is obvious that this additional workload must offer additional benefits to the patient.

In this study, we provided evidence of the need for continuous follow-up of patients with orofacial clefts. The number of therapy recommendations given by different specialists from the most relevant disciplines strongly indicates that the examinations should not be performed by one healthcare professional alone. From the age of three, more than half of the patient group received a therapy recommendation. Within each specialty, certain time frames with intense treatment demand became apparent, which partially overlapped. We found that, in our follow-up program, nearly all therapy recommendations were statistically attached to a certain age (Table 1). For all the specialties, we must consider that the age distribution of recommendations might vary to a certain extent between treatment centers, depending on the clinic’s therapy concept.

We can also infer that the recommendations given by specialty professionals in our multidisciplinary setting changed continuously over the first 16 years of life. Speech therapy was the most commonly recommended up to the age of six, followed by orthodontic treatment. These findings were to be expected because of the drawn-out time span of these therapies [3,13,14,15,16]. This also emphasizes the importance of a close monitoring of and early therapy for speech development disorders [17,18].

The ENT physicians recommended treatment mainly within the first four years, primarily addressing middle ear problems. These are often found even after surgical repair of the palate muscles [19,20]. However, the falling numbers in this field could also be explained by a shift in care to registered ENT doctors outside the clinic.

The evaluation of surgical treatment recommendations showed a multimodal curve over the years. Secondary osteoplasty, rhinoplasty, and orthodontic surgery were mainly responsible for these trends. The high peak of osteoplasty between nine and eleven years of age is due to dentification. This shows the importance of a close follow-up of patients with clefts of the alveolus during second dentition.

The multidisciplinary setting of the consultations additionally provided an advantage by facilitating the coordination of disciplines, as for many issues (e.g., timing of osteoplasty of the jaw, and indication and timing of velopharyngoplasty), the input of more than one specialist is essential.

Not all patients and their parents have the same understanding and appreciation for regular multidisciplinary treatment. Smillie and colleagues showed that socioeconomic status has an influence on the regularity of control follow-up attendance and on adhering to treatment recommendations [21,22]. It should be in the healthcare professional’s interest to overcome this hurdle. A yearly multidisciplinary follow-up would counterbalance this issue and would help to supply professional health treatment for the whole patient group.

We can perceive several limitations in our study. It is retrospective in nature and, therefore, subject to confounding errors. These usual disadvantages associated with a retrospective view on data could only partially be balanced by the fact that the consultation settings of each discipline, the recommendations for follow-up, and the recording methods did not change over time. We also only observed a 15-year period within the ongoing follow-up system, which skewed the age distribution. A prospective study starting with only newborn patients using a longitudinal randomized design would be necessary to produce more solid data on the superiority of multidisciplinary care and the optimal timing and frequency of follow-up examinations.

## 5. Conclusions

In summarizing the different treatment recommendations, we can postulate that there is a high need for the regular follow-up of patients with cleft formations. Some developmental phases seem to be of particular importance for the initiation of treatment protocols within different specialty areas. A multidisciplinary setting is less stressful for young patients and facilitates coordination of the participating specialists. The therapy for and checkup of cleft patients should ideally be concentrated in specialized centers, as in this way, the necessary personal and physical resources can be provided.

## Figures and Tables

**Table 1:** Number of consultations in which one or more therapy recommendations were given, broken down by age in years.

Age at Consultationin Years	Therapy Recommended Number of Consultations (Percentage)	No Therapy Recommended Number of Consultations (Percentage)	Total Number of Consultations Per Age
0	10 (25)	30 (75)	40
1	60 (30.5)	137 (69.5)	197
2	126 (41.9)	175 (58.1)	301
3	197 (62.7)	117 (37.3)	314
4	214 (74.3)	74 (25.7)	288
5	203 (76.6)	62 (23.4)	265
6	186 (72.1)	72 (27.9)	258
7	180 (73.8)	64 (26.2)	244
8	173 (79.7)	44 (20.3)	217
9	169 (85.8)	28 (14.2)	197
10	145 (90.1)	16 (9.9)	161
11	127 (84.1)	24 (15.9)	151
12	111 (83.5)	22 (16.5)	133
13	92 (80)	23 (20)	115
14	86 (81.1)	20 (18.9)	106
15	66 (68.0)	31 (32.0)	97
≥16	272 (70.5)	114 (29.5)	386
Overall	2417 (69.7)	1053 (30.3)	3470

**Table 2:** *p*-values resulting from chi-square test and Fisher’s exact test for the association between age and therapy recommendation.

Therapy Recommendation	*p*-Value
Orthodontic therapy (*n* = 1202)	<0.001
Speech therapy (*n* = 1261)	<0.001
Osteoplasty of the jaw (*n* = 103)	<0.001
Rhinoplasty (*n* = 67)	<0.001
Lip correction (*n* = 32)	<0.001
Orthognathic surgery (*n* = 25)	<0.001
Implantation (*n* = 15)	<0.001
Extraction/tooth exposure (*n* = 119)	<0.001
Velopharyngoplasty (*n* = 16)	0.2699
Dental treatment (*n* = 272)	<0.001
ENT treatment (*n* = 139)	<0.001
Correction of frenula (*n* = 10)	0.2224
Closure of oronasal fistula (*n* = 9)	0.923
Occupational therapy/physiotherapy (*n* = 134)	<0.001

## Data Availability

The data presented in this study are available on request from the corresponding author.
